# Affinity- and Format-Dependent Pharmacokinetics of ^89^Zr-Labeled Albumin-Binding VHH Constructs

**DOI:** 10.3390/ph19010120

**Published:** 2026-01-09

**Authors:** Simon Leekens, Peter Casteels, Tom Van Bogaert, Pieter Deschaght, Veronique De Brabandere, Christopher Cawthorne, Guy Bormans, Frederik Cleeren

**Affiliations:** 1 Laboratory for Radiopharmaceutical Research, Department of Pharmaceutical and Pharmacological Sciences, KU Leuven, 3000 Leuven, Belgium; simon.leekens@kuleuven.be (S.L.);; 2 Sanofi Ghent, 9000 Ghent, Belgium; 3 Department of Imaging and Pathology, Nuclear Medicine and Molecular Imaging, KU Leuven, 3000 Leuven, Belgium

**Keywords:** nanobody, positron emission tomography (PET), half-life extension, DFO*, thiol–maleimide conjugation, pharmacokinetics

## Abstract

**Background/Objectives:** NANOBODY^®^ molecules (VHHs) are attractive vectors for radiopharmaceuticals due to their small size and high target affinity, but rapid clearance and pronounced kidney retention limit their therapeutic applicability. Binding to serum albumin is a widely used strategy to prolong circulation, yet the respective contributions of albumin-binding affinity and molecular format remain insufficiently defined. This study aimed to systematically evaluate how affinity and valency modulate VHH pharmacokinetics. **Methods:** Four monovalent albumin-binding VHHs spanning nanomolar to micromolar affinities and two bivalent constructs were engineered, generated by fusing an albumin-binding VHH to an irrelevant non-binding VHH. All constructs incorporated a site-specific cysteine for DFO* conjugation, enabling uniform zirconium-89 labeling with high radiochemical purity. Pharmacokinetics were assessed in healthy mice using serial blood sampling and positron emission tomography. Blood and kidney exposure were quantified by non-compartmental analysis. **Results:** All albumin-binding constructs showed increased systemic exposure and reduced kidney uptake relative to a non-binding control. Nanomolar-affinity binders reached maximal exposure, and further affinity increases (K_D_ < ~100 nM) did not improve pharmacokinetics, suggesting a threshold. The micromolar binder showed intermediate exposure but still reduced renal retention compared with control. Valency effects were affinity-dependent. They were negligible at high affinity but pronounced at low affinity, where bivalency reduced systemic exposure and increased kidney uptake toward control levels. **Conclusions:** Albumin binding enables tuning of VHH pharmacokinetics in an affinity-dependent manner. Above an apparent affinity threshold, pharmacokinetics become format independent, whereas below this threshold, molecular format substantially influences systemic and renal disposition.

## 1. Introduction

Targeted radionuclide therapy (TRT) is a clinically validated treatment modality supported by evidence from the NETTER-1/2 trial (NCT01578239, NCT03972488) for [^177^Lu]Lu-DOTATATE (Lutathera^®^) in gastroenteropancreatic neuroendocrine tumors and the VISION trial (NCT03511664) for [^177^Lu]Lu-PSMA-617 (Pluvicto^®^) in metastatic castration-resistant prostate cancer. These radiopharmaceuticals use a peptide or peptidomimetic molecule as vectors to selectively deliver lutetium-177 to tumor cells. Peptides display fast clearance and high target specificity, making them ideal for diagnostic and therapeutic applications [1]. However, their clinical utility remains constrained by the limited number of tumor-associated receptors amenable to peptide-based targeting, as only few fulfill the stringent requirements for selective, high-affinity, and accessible binding in vivo [2].

Monoclonal antibodies (mAbs) present an alternative strategy, with high-specificity and established clinical use in oncology. However, radiolabeled mAbs have shown only modest efficacy in solid tumors, and their translation to the clinic has proven challenging [3]. Limitations with mAb-based therapeutic radiopharmaceuticals include hematological toxicity [4] due to prolonged blood circulation, low tumor permeability, and hepatic retention of radiolabeled catabolites [5]. As a result, an increasing body of TRT research has shifted towards smaller antibody fragments and engineered derivatives, including NANOBODY^®^ molecules (VHHs), to overcome these barriers [6].

NANOBODY^®^ molecules or VHHs, derived from camelid heavy-chain-only antibodies, represent a promising class of vector molecules with intermediate properties. Compared to mAbs, they retain high target affinity and specificity but are structurally less complex, easier to produce, and highly modular, facilitating engineering and conjugation. Their small size (~15 kDa) enables rapid tissue penetration, which together with fast renal clearance contributes to high-contrast imaging and favorable pharmacokinetics (PK) for diagnostic imaging. This has been demonstrated across a broad range of PET and SPECT radionuclides, including ^68^Ga, ^99m^Tc, ^18^F, ^64^Cu, and ^89^Zr [7,8], with several VHH-based tracers having progressed to clinical trials [9,10,11,12].

For therapeutic purposes, however, the same properties that favor imaging can limit tumor accumulation and reduce the delivered radiation dose. Importantly, multiple studies have demonstrated that radiolabeled VHHs achieve selective uptake in target-expressing tumors in vivo, and several ^177^Lu-, ^131^I-, and ^188^Re-labeled VHHs have been explored in therapeutic settings [13,14]. Nevertheless, tumor uptake is generally moderate and short-lived, while renal accumulation is pronounced. High kidney uptake is inherent to VHH PK due to rapid renal clearance and tubular reabsorption. When VHHs are labeled with residualizing radionuclides, this process leads to renal retention of the radiolabeled catabolites. Although alternative approaches employing non-residualizing radionuclides, such as ^131^I-labeled anti-HER2 VHHs, can reduce renal retention, the kidneys remain the dose-limiting organ for therapy, underscoring the need for PK optimization strategies [15]. Multiple efforts have been made to address these hurdles to improve the therapeutic application of VHHs [16,17].

One approach to enhance the therapeutic performance of VHHs in TRT is to extend their biological half-life. Various strategies have been described, typically aiming to increase molecular size or exploit endogenous recycling pathways to enhance therapeutic potential. These modifications reduce renal clearance and prolong systemic circulation. Common methods include PEGylation, PASylation, glycosylation, and fusion to albumin-binding domains or Fc fragments [18,19,20].

Extensive research has already demonstrated that albumin binding is an effective means to improve tumor accumulation and reduce renal burden. In this study, we evaluate the use of albumin-binding VHHs, which have several key advantages over other strategies. First, their reversible binding to albumin preserves the structural and functional integrity of the VHH, ensuring target engagement without introducing steric hindrance, a risk associated with permanent chemical modifications. Second, this approach leverages an endogenous carrier protein, thereby avoiding the toxicity concerns often associated with non-biodegradable polymers like PEG [21]. Additionally, VHH-based constructs maintain hydrophilicity and favorable biodistribution, unlike other albumin-binding moieties such as Evans Blue or fatty acids, which may increase hydrophobicity and promote hepatobiliary clearance. This approach preserves the intrinsic advantages of VHHs over full-length mAbs, including small size, tumor permeability, and renal elimination [22,23].

**Figure 1:**
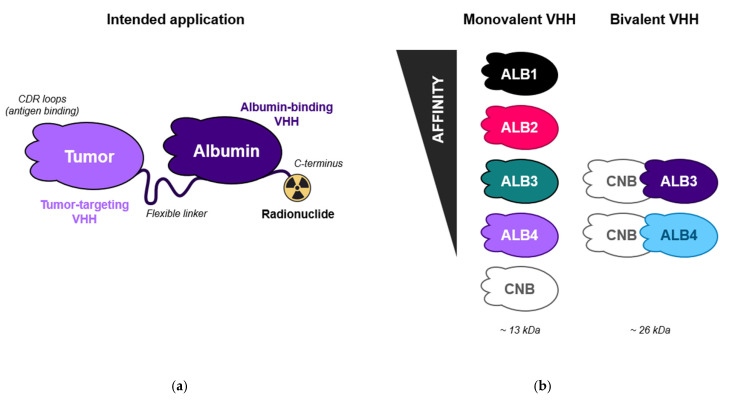
(**a**) Conceptual illustration of the intended application: albumin-binding VHH constructs are used to modulate the PK of a tumor-targeting VHH-based radiopharmaceutical, enabling prolonged systemic circulation and optimized tumor accumulation. (**b**) Schematic representation of the VHH constructs evaluated in this study. All constructs are shown with their N- and C-termini oriented left to right. Bivalent formats include a non-targeting, control VHH (CNB) at the N-terminus followed by an albumin-binding VHH (ALB) at the C-terminus, joined by a flexible GS-linker. Each construct is equipped with a C-terminal cysteine to allow site-specific conjugation for radiolabeling.

The optimal biological half-life for a radiopharmaceutical depends on multiple factors, including application (imaging or therapy), tumor type, target biology, microenvironment, and the physical half-life of the radionuclide [24]. Tailoring the PK profile is therefore crucial. In principle, this can be achieved by modulating the binding affinity of the albumin-binding VHH, offering a tunable and adaptable platform for radiopharmaceutical optimization (Figure 1a). However, a 2020 study by van Faassen et al. [25] highlighted that affinity at neutral pH alone does not fully predict half-life. More specifically, retention of affinity at endosomal pH, which supports FcRn-mediated recycling, is also a key determinant. Building on these insights, we selected albumin binders that maintained their affinity under both neutral and early endosomal pH (pH 6) conditions.

To this end, we designed a panel of VHH constructs with varying affinities for serum albumin, including four monovalent and two bivalent binders (Figure 1b). All constructs contained a single albumin-binding VHH. In the bivalent format, this was fused to an irrelevant, non-binding control VHH (CNB) to mimic a common therapeutic format. The bivalent constructs were specifically included to investigate whether C-terminal positioning of an albumin-binding VHH influences PK, as albumin-binding VHHs are generally combined with a disease-targeting construct [26]. This strategy enabled us to systematically assess the impact of both albumin affinity and format on PK behavior.

To evaluate the in vivo profile of these constructs, they were site-specifically labeled with zirconium-89 using conjugation with DFO*, an octadentate and improved chelator based on deferoxamine [27]. Zirconium-89 is a positron-emitting radionuclide with a physical half-life of 3.3 days, allowing longitudinal positron emission tomography (PET) imaging and quantitative analysis over a sufficiently large time window, in contrast to shorter-lived PET isotopes such as ^68^Ga, ^18^F, and ^64^Cu [28]. ^124^I represents an alternative longer-lived PET isotope (t_1/2_ = 4.2 d) [29]; however, as a non-metal radionuclide it relies on covalent iodination chemistry, which is less directly aligned with the coordination chemistry employed for most therapeutic radiometals such as ^177^Lu and ^225^Ac. Accordingly, ^89^Zr was selected as a quantitative PET tool to support longitudinal characterization of biodistribution and PK relevant to therapeutic design, rather than as the intended radionuclide for diagnostic implementation.

All constructs featured a C-terminal engineered free cysteine, enabling thiol–maleimide chemistry for conjugation of the bifunctional chelator [30]. To our knowledge, this is the first report of optimized site-specific zirconium-89 labeling of VHHs using DFO*–maleimide, providing a robust labeling strategy for the development of half-life-extended VHH-based radiopharmaceuticals.

This study details the labeling strategy, binding kinetics, and a comprehensive in vivo PK characterization of ^89^Zr-labeled albumin-binding VHH constructs aimed at improving radiopharmaceutical performance.

## 2. Results and Discussion

### 2.1. VHH Constructs and Surface Plasmon Resonance

To investigate the influence of albumin-binding affinity on PK, we generated a panel of VHH constructs with varying binding strengths to mouse serum albumin (MSA) and differences in format (Figure 1b). These VHHs were selected from known albumin binders that retained FcRn interaction, ensuring that differences in PK would primarily reflect affinity and format. A broad range of affinities was included to capture biologically meaningful differences in PK. Since albumin is highly abundant in serum, small differences in dissociation equilibrium constant (K_D_) may not result in observable effects in vivo. The available well-characterized VHHs covered a K_D_ range of approximately 1–1000 nM (ALB1, ALB2, ALB3, ALB4).

Molecular format was included as a second design parameter, since albumin-binding constructs for PK modulation are typically fused with disease-targeting constructs [26]. To assess this effect, two bivalent constructs based on ALB3 and ALB4 were included: CNB-ALB3 and CNB-ALB4, respectively. The non-targeting VHH (CNB) at the N-terminus served as a decoy construct to emulate the bivalent architecture without introducing receptor binding. Finally, a monovalent non-binding control (CNB) was included as a baseline comparator for constructs designed to extend biological half-life.

The panel of VHH constructs required biophysical characterization to obtain information on binding kinetics. Binding strength was assessed at physiological pH (7.4) and late-endosomal pH (~6.0), where endogenous albumin is recycled via FcRn [31,32]. Maintaining albumin binding at lower pH is critical, since a complete loss of affinity would result in the VHH construct failing to recycle and instead being degraded, thus compromising its intended biological half-life extension [25]. For this purpose, surface plasmon resonance (SPR) was used as a robust and established technique for comparing binding kinetics. The setup allowed for direct comparison of K_D_ across constructs in a consistent and reproducible manner.

Kinetic analysis revealed a clear spread of binding affinities to MSA at pH 7.4 (Table 1). The high-affinity monovalent constructs ALB1, ALB2, and ALB3 showed K_D_ values of 4.33 nM, 12.6 nM, and 115 nM, respectively. CNB-ALB3 showed similar binding strength to ALB3, with a K_D_ of 177 nM. ALB4 and CNB-ALB4 showed sensorgrams that fell outside the detection limit of the SPR instrument. K_D_ values were estimated but are only indicative: ALB4 (~2.53 µM) and CNB-ALB4 (~6.34 µM). These SPR data confirmed a broad range of binding affinities. Obtaining precisely defined kinetic parameters was not the aim of this study, therefore, the indicative results for the lower-affinity binders were considered sufficient and were reported as micromolar K_D_ values. Measurements performed at pH 6.0, reflecting late endosomal conditions, showed that the VHH constructs retained binding strength, with K_D_ values remaining in the same order of magnitude as those observed at pH 7.4. Based on this biophysical characterization, all constructs were advanced to in vivo PK evaluation.

### 2.2. Site-Specific Labeling of VHHs with Zirconium-89

We opted to site-specifically label the panel of VHH constructs with zirconium-89 to ensure accurate PK evaluation through longitudinal PET/CT. Site-specific labeling was preferred over random labeling to improve product homogeneity, which is critical when comparing the PK of multiple constructs. To achieve this, all constructs were engineered with a C-terminal cysteine to enable thiol–maleimide conjugation, a well-established, rapid, and reliable chemical reaction [30,33]. The VHH constructs used in this study contained no other solvent-exposed cysteines, resulting in selective conjugation at the C-terminus, away from the antigen binding site. Random conjugation can potentially modify residues involved in antigen binding, thereby affecting functionality.

**Figure 2:**
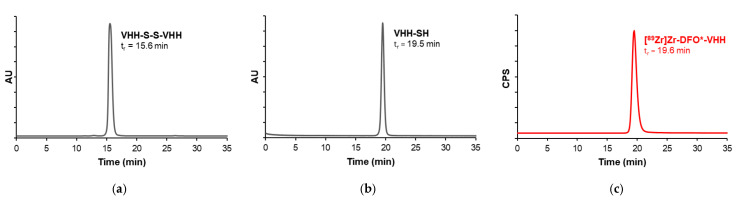
Representative analytical SEC profiles of ALB2. All chromatograms were normalized such that the principal peak height is consistent across panels, enabling qualitative comparison of elution profiles. (**a**) UV chromatogram (280 nm) of the starting VHH material in PBS, showing the oxidized dimeric species (VHH-S-S-VHH) with a retention time of 15.6 min. (**b**) UV chromatogram (280 nm) after reduction with DTT, revealing a shift in retention time to 19.5 min, corresponding to the monomeric VHH (VHH-SH). (**c**) Radiometric chromatogram after radiolabeling and purification of [^89^Zr]Zr-DFO*-VHH, showing a monodisperse peak with a retention time of 19.6 min, consistent with the monomeric VHH. The RCP, as determined by radio-SEC, exceeded 99%, confirming efficient labeling and successful removal of radiolabeled impurities.

A reduction step with dithiothreitol (DTT) was required prior to conjugation, as the engineered C-terminal cysteines formed disulfide-linked VHH dimers under non-reducing conditions (Figure 2a). DTT efficiently reduced these dimers to free thiols (VHH-SH), enabling site-specific conjugation (Figure 2b). However, DTT contains thiol groups that would compete with the maleimide reaction for conjugation sites and thus had to be removed. To achieve this, the sample was subjected to preparative size-exclusion chromatography (SEC) to remove DTT and simultaneously exchange the buffer to phosphate-buffered saline (PBS) containing 0.10 mM tris(2-carboxyethyl)phosphine (TCEP). TCEP is a non-thiol-based reducing agent compatible with maleimide chemistry. Although TCEP is not sufficiently reactive at pH 7.4 in PBS to fully reduce disulfide bonds on its own, it is effective in maintaining already reduced cysteines in their thiol form after DTT removal. The flowthrough containing the monomeric VHH construct was collected and concentrated to 1–2 mg/mL by ultrafiltration then analyzed by SEC and quantified by UV spectroscopy.

Next, 2.5 molar equivalents of DFO*-maleimide were added to the reduced VHH. Although thiol–maleimide conjugation typically occurs within minutes [33], the reaction mixture was incubated for two hours at 37 °C, followed by an overnight incubation at 4 °C. This extended incubation allowed any remaining unconjugated VHH to oxidize. The reaction mixture was then purified by preparative SEC. Oxidized VHH dimers eluted earlier, and the monomeric, conjugated DFO*-VHH was collected. Conjugation yield was determined based on %AUC of the principle peak in the UV-SEC chromatogram. The average conjugation yield was 94%.

The final sample was again concentrated to 1–2 mg/mL by ultrafiltration and quantified by UV spectroscopy. A small aliquot of the conjugate was analyzed by SEC to confirm quality prior to radiolabeling. A single symmetrical peak on SEC confirmed the absence of aggregation or unconjugated material.

The overall recovery yield, accounting for losses during ultrafiltration and preparative SEC, was approximately 60%. On average, ultrafiltration steps resulted in ~85% recovery, while preparative SEC yielded ~90%. Concentration steps were necessary to maintain a protein concentration of 1–2 mg/mL, to support optimal conjugation efficiency and radiolabeling performance. Attempts to improve overall recovery by reducing purification or concentration steps would have been likely to compromise conjugation or radiolabeling efficiency. Therefore, the current protocol represents a good balance between yield and product quality.

Radiolabeling of DFO*-VHH constructs required mild conditions to preserve the stability and functionality of the VHHs. To this end, a labeling protocol was optimized in 0.25 M HEPES buffer (pH 7.2) at 37 °C. A pilot radiolabeling experiment was performed using DFO*-ALB2 as a representative construct, aiming to identify suitable DFO*-VHH concentration and incubation time. Based on these findings, all VHH constructs were subsequently radiolabeled using the optimized protocol.

**Figure 3:**
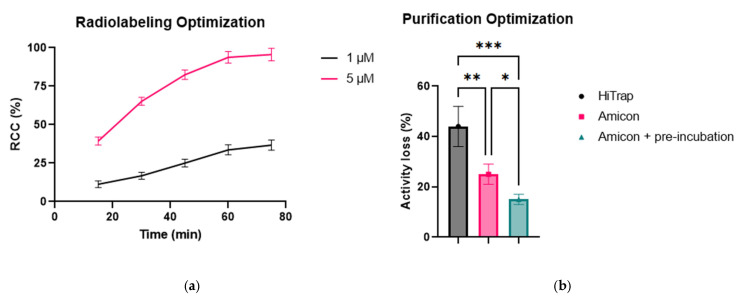
Radiolabeling optimization and purification. (**a**) RCC of [^89^Zr]Zr-DFO*-ALB2 over time under two labeling conditions: 1 µM and 5 µM of DFO*-ALB2 (mean ± SD, *n* = 3). RCC was determined by radio-iTLC followed by autoradiography. (**b**) Optimization of radiolabeling purification using three strategies: HiTrap desalting column, Amicon ultrafiltration, and Amicon ultrafiltration with pre-blocking using unlabeled ALB-H2. Statistical comparison by one-way ANOVA with multiple comparisons confirmed significant differences between all purification strategies (* *p* < 0.05, ** *p* < 0.01, *** *p* < 0.001).

Radiolabeling was optimized and conducted in a total volume of 1 mL, comparing two conditions: 1 µM and 5 µM DFO*-ALB2. Radiochemical conversion (RCC) was monitored over time using radio-instant thin-layer chromatography (iTLC), analyzed via autoradiography. The results showed that DFO*-VHH concentration strongly influenced labeling kinetics (Figure 3a). The 1 µM condition was only able to reach 36 ± 3% RCC after 75 min, whereas the 5 µM group plateaued at 94 ± 4%, with minimal improvement observed between 60 and 75 min. Based on these findings, 5 µM DFO*-VHH and a 60-min incubation time were selected as the standard labeling conditions. Further increases in DFO*-VHH concentration were not pursued, as 5 µM yielded high RCC and efficient radiolabeling.

Different purification strategies were evaluated to remove unbound zirconium-89 from the radiolabeling mixture. Two methods were compared: preparative SEC using HiTrap desalting columns, and ultrafiltration using Amicon filters. In the first approach, the crude labeling mixture was loaded onto four HiTrap columns in series and eluted with formulation buffer at a flow rate of approximately two droplets per minute. Small fractions were collected and activity was measured using a dose calibrator to identify fractions containing the radiolabeled compound. These fractions were then pooled and concentrated by ultrafiltration to reach the desired volume. However, this method was quickly abandoned due to significant activity loss, with 44 ± 8% of the total activity retained on the columns.

In contrast, ultrafiltration using Amicon filters proved more efficient. The crude radiolabeling mixture was concentrated to ~100 µL, resuspended in fresh formulation buffer, and centrifuged again. This washing step was repeated for 4 cycles to effectively remove unbound zirconium-89. Product loss with this method was lower, with 23 ± 4% retained on the filter unit. To further reduce tracer loss, the method was optimized by pre-incubating the Amicon filter unit with 5 nmol of unlabeled VHH. This step was compatible with final formulation, as unlabeled VHH was added to adjust molar activity and protein concentration. Pre-blocking reduced non-specific binding, lowering total activity retention to 15 ± 2% (Figure 3b).

A detailed overview of results of all VHH radiolabeling used for the in vivo study is provided in Table 2. An average RCC of 88 ± 6% was obtained. After purification, all groups showed a radiochemical purity (RCP) > 99%, confirmed by radio-SEC as a single peak at the elution time of the corresponding VHH (Figure 2c and Figure S1). The UV signal of the principal peak was integrated to calculate the molar amount of VHH in the purified radiolabeled sample, using a three-point calibration curve generated from the corresponding non-labeled VHH. The radiolabeled samples were then spiked with additional non-labeled VHH to reach the desired injection dose of 5 mg/kg total VHH.

Injecting only radiolabeled material without adjusting for total protein mass would result in a low mass-dose injection, which can lead to biased PK profiles due to rapid uptake by high-capacity binding in non-target organs such as the liver, spleen, and kidneys. This behavior is observed with radiolabeled biological vectors and typically requires adjusting the molar activity or co-injecting non-labeled protein. The addition of unlabeled VHH improves tracer retention in blood and ensures a more reproducible evaluation of PK [34]. No formal dose optimization was performed; 5 mg/kg was selected as a practical and sufficiently high dose to minimize non-specific clearance.

In vitro stability of the purified radiolabeled VHHs was evaluated in formulation buffer at 4 °C, using radio-iTLC. All constructs showed high stability over time, with >95% of the tracer remaining intact up to 72 h. Stability is displayed in Figure S2 as the percentage of intact radiolabeled compound over time. No noticeable degradation or release of free zirconium-89 was observed during the analysis period, indicating that the DFO* conjugation and radiolabeling protocol resulted in stable products suitable for in vivo application.

### 2.3. In Vivo Evaluation in Healthy Mice

**Figure 4:**
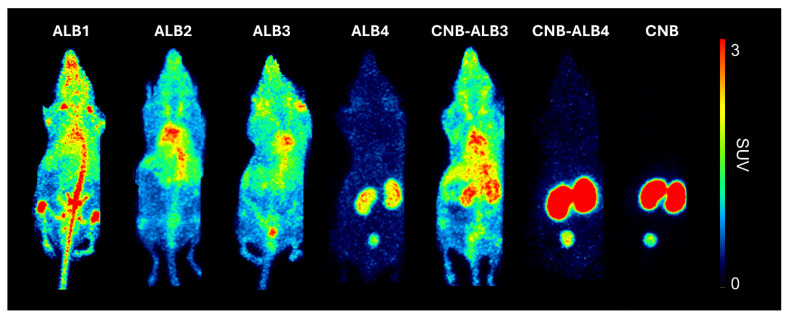
Maximum intensity projection PET images of representative mice at 24 h pi, SUV 0–3 scale. Higher affinity binders ([^89^Zr]Zr-ALB1, [^89^Zr]Zr-ALB2, [^89^Zr]Zr-ALB3, [^89^Zr]Zr-CNB-ALB3) showed systemic signal consistent with extended circulation. No off-target uptake was observed. Lower affinity ([^89^Zr]Zr-ALB4, [^89^Zr]Zr-CNB-ALB4) and non-binding ([^89^Zr]Zr-CNB) constructs showed minimal background and high renal accumulation.

The in vivo evaluation of ^89^Zr-labeled VHH constructs was performed in healthy female NMRI mice (5–6 weeks) that received a single bolus injection with an average activity of 2.41 ± 0.59 MBq and a VHH dose of 4.30 ± 1.11 mg/kg (Table S1). All animals underwent PET imaging, followed by blood collection at each timepoint to generate time–activity curves (TACs) (Figure 4).

Visual inspection of the PET images suggested affinity-related differences in biodistribution. At 24 h pi, the higher-affinity albumin binders (ALB1, ALB2, ALB3, CNB-ALB3), showed widespread vascular and tissue uptake, whereas the lower-affinity binders (ALB4, CNB-ALB4) and the non-binding control (CNB) showed limited systemic uptake and pronounced renal signal (Figure 4).

**Figure 5:**
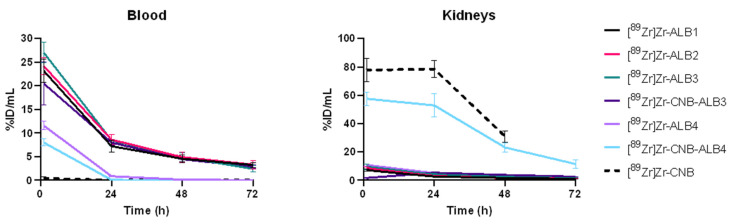
Blood and kidney time-activity curves (1–72 h pi). Blood TACs, were derived from serial blood sampling. Data are shown as mean ± SD. Kidney TACs were derived from PET-based kidney VOIs. For the non-binding control ([^89^Zr]Zr-CNB), PET imaging was performed up to 48 h pi due to rapid clearance, whereas all albumin-binding constructs were imaged up to 72 h.

Inspection of the blood TACs indicated three distinct kinetic profiles (Figure 5). High-affinity albumin-binding constructs ([^89^Zr]Zr-ALB1, [^89^Zr]Zr-ALB2, [^89^Zr]Zr-ALB3, and [^89^Zr]Zr-CNB-ALB3) clustered closely and displayed sustained systemic activity over time. An intermediate profile was observed for the lower-affinity binders [^89^Zr]Zr-ALB4 and [^89^Zr]Zr-CNB-ALB4, which showed partial half-life extension with faster clearance compared to the high-affinity group. Lastly, [^89^Zr]Zr-CNB exhibited rapid clearance, consistent with the PK of an unmodified VHH. Kidney TACs showed highest uptake for [^89^Zr]Zr-CNB, while all albumin-binding constructs exhibited markedly reduced renal activity, except for [^89^Zr]Zr-CNB-ALB4, whose TAC lay right below that of the control.

**Figure 6:**
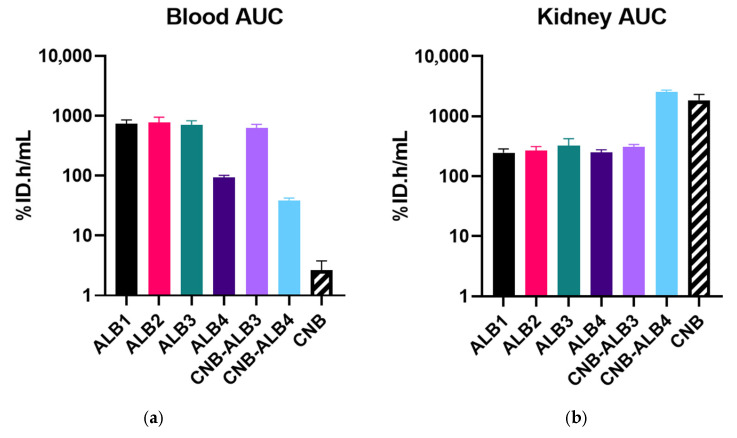
PK parameters of the VHH constructs in healthy NMRI mice. (**a**) Systemic exposure, expressed as blood AUC (%ID.h/mL), was highest for ALB1, ALB2, and ALB3, followed by a marked drop for ALB4 and CNB-ALB4, and minimal exposure for CNB. (**b**) Kidney exposure, expressed as kidney AUC (%ID.h/mL), was markedly reduced for all monovalent albumin-binding constructs compared with CNB, except for the bivalent CNB-ALB4, which exhibited kidney retention similar to CNB. All y-axes are presented on a log_10_-scale. Bars represent geometric mean ± geometric SD (n = 3–4). All measurements were performed up to 72 h pi, except kidney exposure for CNB, which was quantified up to 24 h pi.

To quantify these qualitative observations, blood sample data and kidney VOI-derived PET data were analyzed by non-compartmental analysis (NCA), focusing on AUC (t_0_–t_∞_ or t_0_–t_last_) as the primary parameter to characterize systemic and renal PK (Figure 6). The individual blood and kidney concentration data are provided in Tables S2 and S3, the NCA results in Table S4, and the pairwise statistical comparisons in Table S5.

The baseline control (CNB) exhibited the lowest systemic exposure (2.8 ± 1.3%ID.h/mL), consistent with the PK profile of a non-half-life-extended VHH undergoing rapid renal clearance due to its small molecular size and lack of albumin interaction. In contrast, all albumin-binding constructs showed significantly higher blood AUC values (all *p* < 0.0001), confirming successful biological half-life extension through albumin engagement. The magnitude of this increase, ranging from approximately 15-fold for the fastest clearing albumin binder to nearly 300-fold for the slowest clearing constructs, reflects the tunable nature of albumin-binding VHHs.

To investigate the observed variation in blood PK, we first examined the influence of albumin-binding affinity by comparing constructs within the same molecular format. Among the monovalent albumin-binders, the stronger binders were ^89^Zr-labeled ALB1-3, which displayed statistically indistinguishable blood exposure levels (all *p* > 0.9999) with AUC values between 711 and 780%ID.h/mL. Relative to [^89^Zr]Zr-CNB, these corresponded to an approximate 280-fold higher systemic exposure (95% CI 163–474). The weakest binder, [^89^Zr]Zr-ALB4, exhibited a smaller but significant increase (92.9 ± 1.1%ID.h/mL; 35-fold vs. [^89^Zr]Zr-CNB; 95% CI 20.5–59.7). A similar pattern was observed among the bivalent constructs, where [^89^Zr]Zr-CNB-ALB3 showed significantly higher exposure than [^89^Zr]Zr-CNB-ALB4 (*p* < 0.0001).

Although ALB1-3 spanned approximately two orders of magnitude in albumin-binding affinity, the indistinguishable blood exposure in vivo indicates the presence of an apparent affinity threshold. Increases in binding strength beyond K_D_~100 nM (ALB3) did not further increase systemic exposure.

Next, we evaluated whether the construct format further influenced systemic PK. In the higher-affinity tier ([^89^Zr]Zr-ALB3 vs. [^89^Zr]Zr-CNB-ALB3, *p* = 0.9998), format had no measurable effect. However, for the lowest affinity binders ([^89^Zr]Zr-ALB4 vs. [^89^Zr]Zr-CNB-ALB4, *p* = 0.0013), the bivalent construct displayed a smaller degree of half-life extension, corresponding to a 15-fold increase relative to [^89^Zr]Zr-CNB (95% CI 8.52–24.9). Consequently, [^89^Zr]Zr-CNB-ALB4 exhibited the lowest blood exposure of all albumin-binding constructs tested.

Renal exposure displayed an inverse pattern, consistent with the kidney-protective effect of albumin engagement [20]. [^89^Zr]Zr-CNB showed the highest kidney exposure (1860 ± 1.09%ID.h/mL), significantly exceeding all other binders (all *p* < 0.0001) except [^89^Zr]Zr-CNB-ALB4 (*p* = 0.3184). Among monovalent albumin binders, affinity had no significant effect on renal exposure (all *p* ≥ 0.6753). Overall, these constructs displayed a pooled seven-fold reduction in kidney AUC relative to [^89^Zr]Zr-CNB (95% CI 4.8–9.3). Format had no effect at higher affinity ([^89^Zr]Zr-ALB3 vs. [^89^Zr]Zr-CNB-ALB3) but did at low affinity, where [^89^Zr]Zr-CNB-ALB4 showed a partial loss of the kidney-protective effect observed for the other albumin-binding constructs. Because [^89^Zr]Zr-CNB was only sampled up to 24 h pi, the [^89^Zr]Zr-CNB vs. [^89^Zr]Zr-CNB-ALB4 comparison was re-evaluated over a matched 0–24 h integration window, in which CNB-ALB4 showed slightly lower kidney exposure than [^89^Zr]Zr-CNB (1127 ± 1.029%ID.h/mL vs. 1373 ± 1.085%ID.h/mL, *p* = 0.0109). This suggests that molecular format does influence kidney uptake in lower-affinity settings while still retaining partial kidney protection.

To place these findings in context, previous studies have emphasized the role of pH-dependent albumin binding in maintaining FcRn-mediated recycling. Van Faassen et al. (2020) [25] demonstrated that retaining affinity at endosomal pH is critical for albumin-mediated half-life extension. However, their dataset was heterogeneous, combining albumin-binding domains, VHHs, peptides, and mAbs across multiple studies, which limited a quantitative correlation between affinity and PK. In this study, we controlled these variables by using VHH scaffolds, a site-specific labeling strategy, a uniform mouse strain, a standardized PK workflow, and albumin binders confirmed to retain affinity at acidic pH. This standardized framework enabled a direct evaluation of how albumin affinity and construct format influence PK behavior in vivo.

Among strategies exploiting FcRn recycling, albumin-binding VHHs appear particularly favorable. A preclinical ^89^Zr-immunoPET study comparing claudin-targeting VHHs fused with either an albumin-binding VHH or an Fc domain showed that Fc-mediated half-life extension similarly prolonged circulation but increased hepatic uptake [35]. This highlights the advantage of albumin-binding VHHs, which offer tunable circulation without introducing Fc-associated liver uptake.

Our data indicate that albumin binding enables stepwise modulation of VHH PK rather than continuous *tuning*. The plateau in systemic exposure among high-affinity binders reflect the physiological constraint of MSA turnover (~1–2 days [36]), beyond which slower dissociation cannot further extend biological half-life. Once VHH–albumin binding is sufficiently strong to ensure near-continuous association through rapid rebinding, further increases in affinity do not translate into prolonged systemic exposure. Lowering affinity (as in ALB4) produced an intermediate PK profile characterized by a modest degree of half-life extension, while retaining the kidney-protective effect observed for stronger binders. In contrast to systemic exposure, renal handling appeared predominantly governed by albumin association per se, with limited sensitivity to affinity once binding was achieved. Renal exposure therefore appeared less tunable, as albumin association alone was generally sufficient to reduce kidney accumulation. Notably, [^89^Zr]Zr-CNB-ALB4 showed restored kidney AUC values relative to a non-albumin-binding VHH scaffold. This phenomenon was also observed by van Lith et al. (2022) [37] for VHH constructs bearing albumin-binding domains of both high and low affinities, suggesting that the bivalent architecture may offset renal protection in lower-affinity settings.

From a design perspective, pursuing subnanomolar albumin affinity appears unnecessary in order to achieve maximal biological half-life extension in mice. Within the tested affinity range (K_D_ ≈ 1–200 nM), variations in affinity or format produced indistinguishable PK profiles. Beyond this apparent threshold, micromolar binders may provide a compromise between circulation time and tissue penetration in therapeutic applications. In such cases, the effect of construct valency should be evaluated explicitly, as both affinity and format influence the PK of lower-affinity albumin binders. Translation to humans, where albumin circulates far longer (~19 days), may shift the affinity threshold and would therefore require dedicated evaluation [23].

While the present study was designed to elucidate the mechanistic impact of albumin-binding affinity and format on VHH PK, translation to a therapeutic radiopharmaceutical context introduces additional considerations. In a therapeutic setting, prolonged systemic exposure inevitably increases radiation burden to healthy tissues, including radiosensitive compartments such as bone marrow. As such, half-life extension represents a balancing act between enhancing tumor accumulation, reducing renal dose, and limiting hematological toxicity.

In this context, the choice of radionuclide becomes critical. Prolonged circulation may be less compatible with β^−^-emitting radionuclides due to their relatively long particle range and associated cross-fire irradiation of non-target tissues. In contrast, α-particle emitters and Auger electron emitters, which deposit their energy over much shorter path lengths, may be better suited to half-life-extended VHH constructs [38]. Nevertheless, increased systemic exposure remains a potential liability regardless of radionuclide choice. Ultimately, dedicated dosimetry and toxicity studies in tumor-bearing models will be required to fully assess the therapeutic feasibility and safety window of albumin-binding VHH-based radiopharmaceuticals.

## 3. Material and Methods

### 3.1. Reagents

Chemicals and solvents were acquired from Merck (Bornem, Belgium), Acros Organics (Geel, Belgium), Fluka (Bornem, Belgium), or Thermo Fisher Scientific (Doornik, Belgium), unless stated otherwise. All buffers used during labeling were Chelexed (Chelex 100 sodium form), 50 g/L, 30 min stirring, and filtered (0.45 µm, polyamide filter; Sartorius, Stedim Biotech, Göttingen, Germany). VHH constructs were expressed and purified at Sanofi (Ghent, Belgium).

### 3.2. VHH Panel Design

A panel of albumin-binding VHHs was designed and expressed to systematically evaluate the effects of affinity and molecular valency on PK in vivo. The panel included four monovalent constructs (ALB1, ALB2, ALB3, ALB4), covering a broad range of affinity for MSA. To assess the impact of valency, two bivalent constructs were generated by fusion of an albumin-binding domain to a non-binding VHH scaffold (CNB), yielding CNB-ALB3 (using ALB3 as albumin binder) and CNB-ALB4 (using ALB4 as albumin binder). The CNB building block does not bind to any target in healthy NMRI mice and was used to emulate the bivalent molecular format without introducing specific target interactions. The monovalent CNB was included as a non-albumin binding baseline control.

### 3.3. Synthesis and Radiolabeling

VHH constructs (2.5 mg; 12 mg/mL in PBS, pH 7.4) were reduced in 10 mM DTT in PBS, pH 7.4, for 2 h at room temperature while shaking at 350 RPM (Eppendorf ThermoMixer F2.0; Merck, Darmstadt, Germany). Samples were buffer-exchanged into 0.10 mM TCEP in PBS (pH 7.4) using preparative SEC on a Superdex75 Increase 100/300 GL (Merck; Darmstadt, Germany) with an isocratic mobile phase of PBS, pH 7.4, at 0.750 mL/min, on an Agilent 1100 Series HPLC Value System equipped with a variable wavelength detector (Agilent G1314A; Santa Clara, CA, USA). If the volume of the eluted fractions exceeded 1 mL, a concentration step was performed using Amicon Ultra centrifugal filters (Merck, Darmstadt, Germany; MWCO 3 kDa for monomeric constructs and 10 kDa for bivalent constructs).

Reduced VHH constructs were conjugated to DFO*–maleimide (ABX, Radeberg, Germany) using a 2.5-fold molar excess (10 mM in DMSO) for 2 h at room temperature, 350 RPM. DMSO content was kept below 5% (*v*/*v*) of the total reaction volume. Unreacted thiols were capped with an excess of N-maleoyl-β-alanine (200 nmol). The reaction was incubated overnight at 4 °C, followed by purification by preparative SEC as described above. VHH purity and concentration was assessed spectroscopically at 260 and 280 nm (Nanodrop OneC, Thermo Fisher Scientific, Doornik, Belgium).

[^89^Zr]Zr-oxalate (in 1 M oxalic acid) was obtained from BV Cyclotron VU (Amsterdam, The Netherlands) and used to label DFO*-VHH constructs to achieve a molar activity of 10 MBq/nmol. Briefly, 2.5 nmol of DFO*-VHH (from a 1 mg/mL stock in PBS, pH 7.4) was diluted in 500 µL of HEPES buffer (0.5 M, pH 7.2). To this, 45 µL of 2 M Na_2_CO_3_ was added to neutralize the oxalic acid from the ^89^Zr solution. The reaction volume was adjusted to 900 µL with 0.9% (*m*/*v*) NaCl saline, after which 100 µL of [^89^Zr]Zr-oxalate in 1 M oxalic acid was added. When less than 100 µL was used, the missing volume of oxalic acid was added prior to [^89^Zr]Zr-oxalate addition to maintain constant pH. The reaction was incubated for 1 h at 37 °C, 350 RPM (ThermoShaker, Thermo Fisher Scientific), then purified using ultrafiltration (MWCO 3 kDa for monomeric and 10 kDa for bivalent constructs) and washed four times with formulation buffer (5 mg/mL ascorbic acid in PBS, pH 7.4).

Radiolabeled constructs were analyzed by radio/UV-SEC using the setup described above, equipped with a GABI* radioactivity flow detector (Elysia-Raytest; Liège, Belgium). A 20 µL aliquot of purified product was injected to determine RCP and VHH concentration. RCP was calculated from the radiometric chromatogram as the %AUC corresponding to the VHH peak identified in the UV trace. VHH concentrations were determined by integrating the UV peak and converting to mass using the calibration curve of the corresponding non-labeled VHH construct. This construct was prepared by capping the thiol groups of the reduced VHH with N-maleoyl-β-alanine under identical conjugation conditions and is subsequently referred to as “non-labeled VHH”. It was used to spike the radiolabeled compound to adjust the molar activity and final injection dose. All injections were prepared to deliver 3 MBq, 5 mg/kg VHH per mouse, in a total volume of 100 µL.

### 3.4. Binding Kinetic Experiments

The kinetic rate constants and affinity constants (k_on_, k_off_, and K_D_) of the tested VHH panel for MSA were determined via SPR on a ProteOn XPR36 instrument (Biorad, Lokeren, Belgium) at 25 °C using HBS-EP+ as running buffer. Approximately 750 RUs of MSA was immobilized on a GLC sensorchip (Biorad, Lokeren Belgium) using a ProteOn Amine Coupling Kit (Biorad) according to the manufacturer’s instructions. As an analyte, VHHs were injected according to a multicycle kinetics protocol at concentrations ranging between 4.12 to 1000 nM, followed by a dissociation phase over 10 min. Data were analyzed with Biacore insight evaluation software (version 5, Cytiva, Hoegaarden, Belgium). The kinetic rate constants (k_on_ and k_off_) were calculated by fitting the sensorgrams via the Langmuir 1:1 interaction ligand binding model. The equilibrium dissociation constant K_D_ was calculated as the k_off_/k_on_ ratio.

### 3.5. In Vitro Evaluation

The stability of ^89^Zr-DFO*-VHH constructs was evaluated in formulation buffer (5 mg/mL ascorbic acid in PBS, pH 7.4) at 4 °C, 350 RPM. The assay was performed in triplicate using 100 µL of radiolabeled tracer (~20 kBq/µL) diluted in 900 µL of formulation buffer. At 4, 24, 48, and 72 h, 3 µL aliquots were spotted onto iTLC-SG strips (glass microfiber chromatography paper impregnated with silica gel; Agilent Technologies, Folsom, CA, USA) and developed in 2 mM citrate buffer (pH 5.0). Autoradiography was performed using europium-activated phosphor storage screens (super-resolution screen, Perkin Elmer, Waltham, MA, USA), and signal was recorded using the Cyclone Plus system (Perkin Elmer). Quantification was carried out with OptiQuant software (version 5.0, Perkin Elmer). Stability was reported as the percentage of radioactivity retained at the origin (Rf ≈ 0), corresponding to intact ^89^Zr-DFO*-VHH. Free ^89^Zr species migrated with an Rf of approximately 0.9.

### 3.6. In Vivo Evaluation in Healthy Mice

Female NMRI mice (5–6 weeks old) were purchased from Inotiv (Venray, The Netherlands) and housed in individually ventilated cages under controlled temperature (approximately 22 °C) and humidity, with a 12:12 h light–dark cycle and access to food and water ad libitum.

Mice received a bolus tail vein injection of approximately 3 MBq ^89^Zr-DFO*-VHH (5 mg/kg VHH). Whole-body PET imaging was performed at 1, 24, 48, and 72 h pi using the β-cube PET scanner (Molecubes, Ghent, Belgium). Animals were anesthetized with isoflurane (5% for induction, 2.5% for maintenance) in oxygen at a flow rate of 1 L/min and positioned head-first prone in the imaging cell (Molecubes) prior to scanning. Respiration and body temperature were monitored throughout the imaging session.

Following PET acquisition, CT scans were performed for anatomical co-registration using the X-cube CT scanner (Molecubes) with the ‘General Purpose’ protocol: 50 kVp, 480 exposures, 85 ms/projection, 100 μA tube current, and a 60 s rotation time. PET and CT images were fused and scaled to kBq/mL or SUV. Kidney uptake was quantified using manually defined VOIs based on fused PET/CT data. Coronal maximum intensity projections were generated to visualize tracer distribution. Image analysis was performed using PFUS v4.0 (PMOD Technologies GmbH, Zurich, Switzerland).

Immediately following each CT scan, blood samples were collected and measured using a Wizard^2^ γ-counter (2480-0010, PerkinElmer, Waltham, MA, USA). Radioactivity concentration in blood was expressed as %ID/g.

### 3.7. Non-Compartmental Analysis

To explore the pharmacokinetic behavior of the VHH constructs, systemic and renal exposures were quantified. Blood and kidney activity concentrations were normalized to the injected dose and expressed as %ID/mL. For ex vivo blood data, values calculated as %ID/g were interpreted as %ID/mL, assuming a tissue density of approximately 1 g/mL, consistent with standard practice in small-animal biodistribution studies. Blood and kidney TACs were analyzed in R (version 4.5.1) using the ncappc package (version 1.0.0). Data handling and visualization were performed with tidyverse (version 2.0.0), dplyr (version 1.1.4), and ggplot2 (version 4.0.0), following the workflow described by Dunvald et al. (2022) [39]. NCA was conducted using the linear-up/log-down trapezoidal rule to derive AUC. The apparent terminal elimination rate constant (λ_z_) was estimated from the log-linear terminal phase, and total exposure was expressed as AUC_0–∞_, calculated as AUC_last_ + C_last_/λ_z_. For constructs with rapid clearance, where λ_z_ could not reliably be estimated, exposure was calculated as AUC_last_. This applied to the blood TACs of CNB and the kidney TACs of ALB4. All other groups were extrapolated to infinity.

### 3.8. Statistical Analysis

All AUC data were log_10_-transformed prior to statistical analysis to stabilize variance and approximate normality. Group comparisons were performed by one-way ANOVA, followed by predefined pairwise tests between biologically relevant groups: each construct versus CNB, comparisons among monovalent albumin binders, among bivalent binders, and between monovalent and bivalent formats within the same affinity tier. A Šídák’s correction was applied to adjust for multiple comparisons. Statistical significance was set at α = 0.05. Results are presented as geometric means with geometric standard deviation factors, and group differences are expressed as geometric mean ratio with corresponding 95% CI. When constructs showed statistically similar effects relative to CNB, a pooled geometric mean ratio was calculated. Statistical testing was performed using GraphPad Prism (v9.0, GraphPad Software, San Diego, CA, USA).

## 4. Conclusions

This study establishes a robust, site-specific protocol for ^89^Zr-labeling of VHH constructs using DFO*–maleimide, yielding highly stable tracers with excellent RCP suitable for longitudinal PET/CT studies. Applying this protocol, we systematically characterized the PK of ^89^Zr-labeled albumin-binding VHHs, demonstrating that both binding affinity and construct format modulated systemic exposure and renal handling in an affinity-dependent manner. These findings provide a framework for rationally tuning the PK of VHH-based radiopharmaceuticals through controlled VHH–albumin binding.

## Figures and Tables

**Table 1:** Binding kinetics of VHH constructs to mouse serum albumin.

Name	Assay pH	k_on_ (M^−1^s^−1^)	k_off_ (s^−1^)	K_D_ (M)
ALB1	6.0	6.68 × 10^5^	2.53 × 10^−3^	3.79 × 10^−9^
	7.4	7.04 × 10^5^	3.05 × 10^−3^	4.33 × 10^−9^
ALB2	6.0	8.25 × 10^5^	9.90 × 10^−3^	1.20 × 10^−8^
	7.4	7.58 × 10^5^	9.58 × 10^−3^	1.26 × 10^−8^
ALB3	6.0	3.10 × 10^5^	3.50 × 10^−2^	1.13 × 10^−7^
	7.4	3.60 × 10^5^	4.12 × 10^−2^	1.15 × 10^−7^
ALB4	6.0	8.44 × 10^4^	5.00 × 10^−1^ *	5.92 × 10^−6^ *
	7.4	1.98 × 10^5^	5.00 × 10^−1^ *	2.53 × 10^−6^ *
CNB-ALB3	6.0	1.73 × 10^5^	3.34 × 10^−2^	1.92 × 10^−7^
	7.4	1.72 × 10^5^	3.05 × 10^−2^	1.77 × 10^−7^
CNB-ALB4	6.0	2.22 × 10^4^ *	8.42 × 10^−2^ *	3.79 × 10^−6^ *
	7.4	1.73 × 10^4^ *	1.10 × 10^−1^ *	6.34 × 10^−6^ *

* Values outside limit of detection of instrument.

**Table 2:** Overview of radiolabeled VHH constructs.

Name	Molecular Weight (Da)	RCC	RCP
ALB1	13,701.0	91% (n = 1)	>99%
ALB2	12,339.6	93 ± 3% (n = 4)	>99%
ALB3	12,314.5	87% (n = 1)	>99%
ALB4	12,265.6	78% (n = 1)	>99%
CNB-ALB3	26,319.1	85% (n = 1)	>99%
CNB-ALB4	26,243.0	83% (n = 1)	>99%
CNB	13,780.0	96% (n = 1)	>99%

## Data Availability

The original contributions presented in this study are included in the article/Supplementary Materials. Further inquiries can be directed to the corresponding author.

## Supplementary Materials

The following supporting information can be downloaded at: https://www.mdpi.com/article/10.3390/ph19010120/s1.

## Abbreviations

The following abbreviations are used in this manuscript:

%AUC	Percentage area under the curve (chromatogram analysis)
AUC	Area under the time–activity curve
AUC_0–last_	Area under the time–activity curve from time zero to the last measurement
AUC_0–∞_	Area under the time–activity curve, from time zero extrapolated to infinity
CT	Computed tomography
DFO*	Octadentate derivative of deferoxamine
DMSO	Dimethyl sulfoxide
DTT	Dithiothreitol
FcRn	Neonatal Fc receptor
HEPES	2-(4-(2-hydroxyethyl)piperazin-1-yl)ethanesulfonic acid
iTLC	Instant thin-layer chromatography
K_D_	Dissociation equilibrium constant
mAb	Monoclonal antibody
MSA	Mouse serum albumin
NCA	Non-compartmental analysis
PBS	Phosphate buffered saline
PET	Positron emission tomography
pi	Post-injection
PK	Pharmacokinetic
RCC	Radiochemical conversion
RCP	Radiochemical purity
SD	Standard deviation
SEC	Size-exclusion chromatography
SPR	Surface plasmon resonance
TAC	Time-activity curve
TCEP	Tris(2-carboxyethyl)phosphine
TRT	Targeted radionuclide therapy
VHH	Variable domain of camelid heavy chain-only antibody
VOI	Volume of interest
