# Supplementary material for: Affinity- and Format-Dependent Pharmacokinetics of 89Zr-Labeled Albumin-Binding VHH Constructs
Source: Pharmaceuticals (Basel). 2026 Jan 9;19(1):120. doi: 10.3390/ph19010120 (PMC12845014; doi:10.3390/ph19010120)
Supplement: Supplementary file 1 [file pharmaceuticals-19-00120-s001.zip › pharmaceuticals-4038469-supplementary.pdf]

SUPPLEMENTARY INFORMATION

Affinity- and format-dependent pharmacokinetics of ^{89}Zr-labeled albumin-binding VHH constructs

Simon Leekens 1, Peter Casteels 2, Tom Van Bogaert 2, Pieter Deschaght 2, Veronique De Brabandere 2,
Christopher Cawthorne 3, Guy Bormans 1 and Frederik Cleeren 1,*

1 Laboratory for Radiopharmaceutical Research, Department of Pharmaceutical and Pharmacological Sciences, KU Leuven, 3000 Leuven, Belgium; simon.leekens@kuleuven.be (S.L.)

2 Sanofi Ghent, 9000 Ghent, Belgium

3 Department of Imaging and Pathology, Nuclear Medicine and Molecular Imaging, KU Leuven, 3000 Leuven, Belgium

* Correspondence: frederik.cleeren@kuleuven.be

SUPPLEMENTARY TABLES

Table S1. Overview of injections

Construct	Number of animals	Weight (g)	Injected dose (MBq)	Injected dose (mg/kg)
ALB1	3	30.5 ± 0.7	1.29 ± 0.37	2.11 ± 0.58
ALB2	3	27.9 ± 0.5	2.49 ± 0.03	4.47 ± 0.03
ALB3	3	26.9 ± 1.5	2.74 ± 0.64	5.08 ± 1.10
ALB4	3	28.4 ± 1.2	2.57 ± 0.14	4.54 ± 0.36
CNB-ALB3	3	29.7 ± 2.2	2.62 ± 0.12	4.42 ± 0.15
CNB-ALB4	3	28.6 ± 1.3	2.82 ± 0.20	4.94 ± 0.32
CNB	4	27.9 ± 1.6	2.05 ± 0.18	3.71 ± 0.54

Table S2. Individual blood concentration data per animal.

Group	Timepoint (h)	Animal 1 (%ID/mL)	Animal 2 (%ID/mL)	Animal 3 (%ID/mL)	Animal 4 (%ID/mL)
**ALB1**	0	45.641%*	47.461%*	45.641%*	
	1.43333	20.392%	24.771%	24.439%	
	24.71667	6.628%	8.696%	6.400%	
	48.76667	4.157%	5.462%	4.088%	
	72.75000	2.966%	3.893%	2.957%	
**ALB2**	0	50.659%*	52.329%*	50.839%*	
	1.36667	25.506%	24.940%	22.199%	
	24.23333	8.947%	9.433%	7.455%	
	52.88333	5.304%	5.698%	3.773%	
	72.35000	3.958%	3.743%	2.264%	
**ALB3**	0	55.371%*	53.505%*	53.908%*	
	1.11667	29.197%	27.139%	24.831%	
	23.65000	8.171%	9.812%	7.468%	
	52.16667	5.453%	5.411%	3.848%	
	71.73333	2.924%	2.636%	1.732%	
**CNB-ALB3**	0	46.685%*	52.521%*	45.496%*	
	1.60000	20.534%	25.127%	15.909%	
	21.28333	7.981%	8.625%	7.395%	
	45.21667	4.651%	5.038%	3.797%	
	69.21667	2.890%	3.201%	2.470%	
**ALB4**	0	48.426%*	52.521%*	50.302%*	
	1.53333	11.019%	11.236%	12.618%	
	21.26667	0.737%	0.898%	0.926%	
	45.18333	0.120%	0.149%	0.157%	
	69.21667	0.035%	0.039%	0.054%	
**CNB-ALB4**	0	50.302%*	52.138%*	47.619%*	
	1.05000	7.779%	8.846%	7.389%	
	21.38333	0.089%	0.102%	0.091%	
	45.26667	0.035%	0.037%	0.035%	
	69.35000	0.019%	0.024%	0.019%	
**CNB**	0	54.526%*	49.092%*	48.591%*	53.107%*
	1.09028	0.227%	0.286%	0.878%	0.318%
	23.85694	0.006%	0.002%	0.003%	0.008%
	47.24028	0.019%	0.014%	0.019%	0.019%
	71.24028	0.025%	0.029%	0.038%	0.010%

**C_0 was not experimentally measured but theoretically derived, assuming 100% of the injected dose is instantaneously distributed within the total blood volume, estimated as 7% of body weight.*

Table S3. Individual kidney concentration data per animal.

Group	Timepoint (h)	Animal 1 (%ID/mL)	Animal 2 (%ID/mL)	Animal 3 (%ID/mL)	Animal 4 (%ID/mL)
ALB1	0.958	6.634%	7.774%	7.188%	
	24.351	2.598%	2.674%	2.570%	
	48.430	1.493%	1.984%	1.381%	
	72.411	0.886%	1.276%	1.056%	
ALB2	0.958	9.324%	8.437%	7.884%	
	23.883	3.171%	3.477%	2.575%	
	52.533	1.608%	1.662%	1.335%	
	72.000	1.147%	1.458%	0.890%	
ALB3	0.958	8.531%	8.490%	11.950%	
	23.602	3.276%	3.575%	5.186%	
	46.973	1.753%	2.160%	3.254%	
	70.962	1.082%	1.271%	1.954%	
CNB-ALB3	1.083	1.809%	1.864%	1.240%	
	20.837	5.680%	5.641%	4.837%	
	44.711	3.813%	4.053%	3.391%	
	68.759	2.625%	2.635%	2.261%	
ALB4	1.067	10.640%	10.030%	11.636%	
	20.838	4.639%	4.972%	5.728%	
	44.677	1.955%	2.001%	2.417%	
	68.775	1.066%	1.123%	1.308%	
CNB-ALB4	1.267	60.776%	52.308%	59.831%	
	21.004	46.624%	61.987%	49.982%	
	45.000	20.129%	26.475%	23.076%	
	68.948	8.402%	14.747%	11.344%	
CNB	0.002	1.181%	0.957%	0.498%	0.859%
	0.006	9.065%	8.535%	5.843%	9.305%
	0.010	16.482%	17.815%	13.864%	18.998%
	0.015	21.429%	23.399%	19.597%	23.955%
	0.025	34.540%	32.993%	28.688%	34.959%
	0.042	49.623%	46.731%	40.828%	49.044%
	0.058	58.785%	56.531%	50.267%	59.120%
	0.075	63.761%	62.431%	56.084%	64.339%
	0.108	71.132%	71.475%	64.889%	69.452%
	0.158	71.206%	76.752%	71.435%	73.556%
	0.208	72.769%	80.979%	74.764%	75.964%
	0.258	71.033%	82.760%	76.255%	75.927%
	0.308	71.072%	83.338%	76.965%	76.104%
	0.375	73.739%	86.398%	77.420%	76.761%
	0.458	72.341%	87.530%	76.145%	77.358%
	0.542	67.892%	83.782%	80.216%	80.205%
	0.625	66.272%	85.395%	82.598%	80.267%
	0.708	65.853%	85.344%	83.219%	79.976%
	0.792	69.888%	87.491%	80.348%	80.857%
	0.875	71.305%	88.461%	77.516%	81.125%
	0.958	70.781%	88.890%	72.389%	79.503%
	3.913	73.282%	85.745%	81.516%	73.659%
	23.507	26.356%	32.145%	35.830%	29.843%

Table S4. Individual and group pharmacokinetic parameters derived from non-compartmental analysis of blood and kidney AUCs.

Matrix	Group	AUC	Log_{10}(AUC)	Geometric mean	GSD
Blood	ALB1	719.23	2.86	792.7	1.139
		918.57	2.96		
		753.85	2.88		
	ALB2	932.88	2.97	825.8	1.214
		914.18	2.96		
		660.30	2.82		
	ALB3	829.02	2.92	744.3	1.167
		797.54	2.90		
		623.52	2.79		
	ALB4	124.09	2.09	133.8	1.076
		134.30	2.13		
		143.73	2.16		
	CNB-ALB3	685.23	2.84	673.4	1.162
		774.93	2.89		
		575.09	2.76		
	CNB-ALB4	61.50	1.79	62.90	1.080
		68.51	1.84		
		59.04	1.77		
	CNB	13.03*	1.11	14.02	1.162
		12.37*	1.09		
		17.38*	1.24		
		13.79*	1.14		
Kidney	ALB1	281.56	2.45	271.4	1.172
		311.34	2.49		
		228.14	2.36		
	ALB2	264.96	2.42	326.4	1.308
		296.83	2.47		
		442.01	2.65		
	ALB3	263.32	2.42	251.6	1.103
		269.02	2.43		
		224.96	2.35		
	ALB4	219.38	2.34	249.0	1.158
		292.54	2.47		
		240.68	2.38		
	CNB-ALB3	292.70*	2.47	312.3	1.100
		298.52*	2.47		
		348.47*	2.54		
	CNB-ALB4	2405.69	3.38	2585	1.066
		2642.72*	3.42		
		2716.44	3.43		
	CNB	1756.77	3.24	1841	1.253
		1405.63*	3.15		
		2425.05	3.38		
		1920.12	3.28		

Shown are individual mouse AUC values ($AUC_{0-\infty}$ or AUC_{0-last} when λ_z could not be estimated), corresponding log_{10}-transformed values used for statistical analysis, and group-level descriptive statistics (geometric mean and geometric standard deviation factor; GSD).
** AUC_{0-last} was reported instead of $AUC_{0-\infty}$ due to unreliable λz estimation ($R^2 < 0.9$ or insufficient terminal data points).*

Table S5. Pairwise statistical comparisons of log_{10}-transformed blood and kidney AUC values.

Statistical test	Mean difference (log_{10})	95% CI (log_{10})		Fold difference (10^{Δ})	95% CI		Summary	Adjusted P value
Blood AUC								
ALB1 vs CNB	2.445	2.213	2.678	279.9	163.2	476.4	****	<0.0001
ALB2 vs CNB	2.463	2.231	2.696	290.7	170.3	497.7	****	<0.0001
ALB3 vs CNB	2.424	2.192	2.656	266.1	155.5	453.7	****	<0.0001
ALB4 vs CNB	1.543	1.311	1.776	35	20.5	59.7	****	<0.0001
CNB-ALB3 vs CNB	2.367	2.135	2.6	232.3	136.3	398.1	****	<0.0001
CNB-ALB4 vs CNB	1.163	0.9305	1.395	14.6	8.52	24.9	****	<0.0001
ALB1 vs ALB2	−0.0179	−0.2662	0.2304	0.96	0.54	1.7	ns	>0.9999
ALB1 vs ALB3	0.0216	−0.2267	0.2699	1.05	0.59	1.86	ns	>0.9999
ALB2 vs ALB3	0.0395	−0.2088	0.2878	1.09	0.62	1.94	ns	>0.9999
ALB1 vs ALB4	0.9022	0.6539	1.151	7.99	4.51	14.2	****	<0.0001
ALB2 vs ALB4	0.9202	0.6718	1.168	8.32	4.7	14.7	****	<0.0001
ALB3 vs ALB4	0.8806	0.6323	1.129	7.59	4.29	13.5	****	<0.0001
ALB3 vs CNB-ALB3	0.0567	−0.1917	0.305	1.14	0.64	2.02	ns	0.9998
ALB4 vs CNB-ALB4	0.3804	0.1321	0.6287	2.4	1.36	4.25	**	0.0013
CNB-ALB3 vs CNB-ALB4	1.204	0.956	1.453	16	9.04	28.4	****	<0.0001
Kidney AUC								
ALB1 vs CNB	−0.8749	−1.072	−0.6778	0.133	0.085	0.21	****	<0.0001
ALB2 vs CNB	−0.8381	−1.035	−0.6410	0.145	0.092	0.229	****	<0.0001
ALB3 vs CNB	−0.7576	−0.9547	−0.5605	0.175	0.111	0.275	****	<0.0001
ALB4 vs CNB	−0.8659	−1.063	−0.6688	0.137	0.087	0.214	****	<0.0001
CNB-ALB3 vs CNB	−0.7787	−0.9758	−0.5816	0.167	0.105	0.263	****	<0.0001
CNB-ALB4 vs CNB	0.1411	−0.0560	0.3382	1.38	0.88	2.18	ns	0.3184
ALB1 vs ALB2	−0.0368	−0.2475	0.1739	0.92	0.57	1.49	ns	>0.9999
ALB1 vs ALB3	−0.1173	−0.3281	0.0934	0.76	0.47	1.24	ns	0.6753
ALB2 vs ALB3	−0.0805	−0.2912	0.1302	0.83	0.51	1.35	ns	0.9676
ALB1 vs ALB4	−0.0091	−0.2198	0.2017	0.98	0.6	1.59	ns	>0.9999
ALB2 vs ALB4	0.0278	−0.1829	0.2385	1.07	0.66	1.73	ns	>0.9999
ALB3 vs ALB4	0.1083	−0.1024	0.319	1.28	0.79	2.09	ns	0.7747
ALB3 vs CNB-ALB3	0.0211	−0.1896	0.2318	1.05	0.65	1.7	ns	>0.9999
ALB4 vs CNB-ALB4	−1.007	−1.218	−0.7963	0.098	0.06	0.16	****	<0.0001
CNB-ALB3 vs CNB-ALB4	−0.9198	−1.130	−0.7091	0.12	0.074	0.195	****	<0.0001

Results of one-way ANOVA with Šídák's multiple comparisons test performed on log_{10}-transformed AUC data. Shown are mean differences on the log_{10} scale, corresponding 95% confidence intervals, back-transformed fold differences (10^Δ) with 95% CIs, adjusted p-values, and significance summaries. Analyses were performed separately for blood and kidney AUC datasets.

SUPPLEMENTARY FIGURES

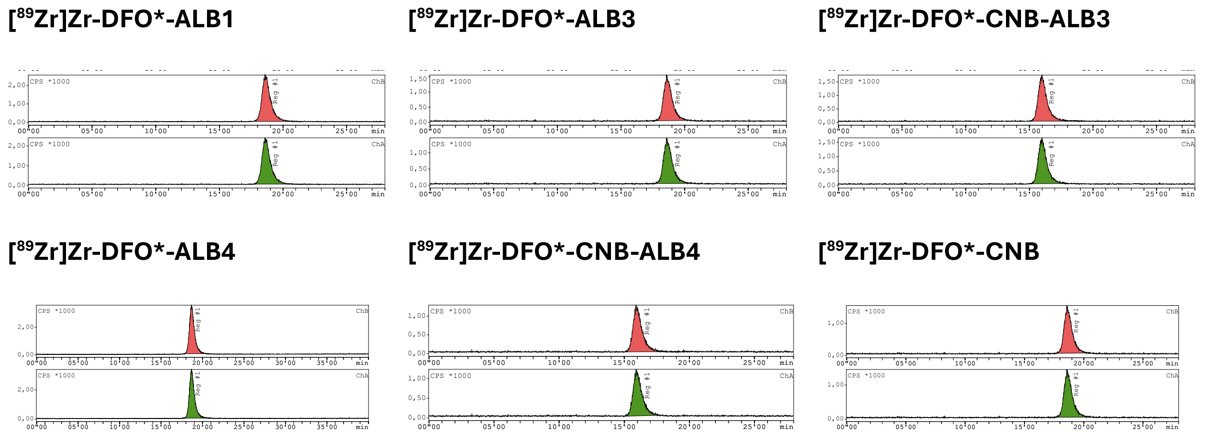

Figure S1. Radio-SEC chromatograms of the remaining radiolabeled VHH constructs after purification. SEC was performed on an Agilent 1100 Series HPLC Value System equipped with a variable wavelength detector (Agilent G1314A) and a GABI* radiometric flow detector (Elysia-Raytest, Liège, Belgium). A Superdex 75 Increase 10/300 GL column (Merck, Darmstadt, Germany) was used with PBS (pH 7.4) as the isocratic mobile phase at a flow rate of 0.750 mL/min. All constructs showed a single-peak radio-chromatogram, corresponding to the expected retention time, indicating RCP > 99%.

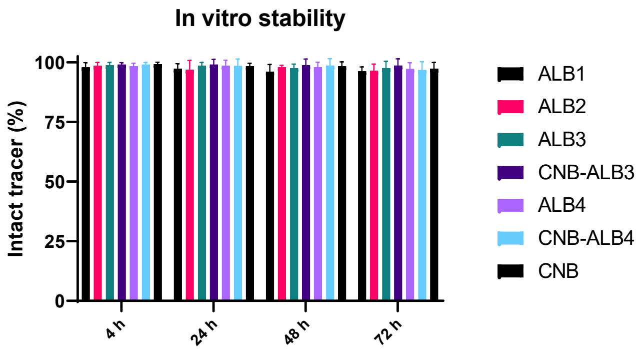

Figure S2. *In vitro* stability of ^{89}Zr-labeled VHH constructs in formulation buffer at 4 °C. Radiochemical stability was assessed by radio-iTLC at various time points up to 72 h. Data represent group means ± SD (n = 3). All constructs maintained sufficient radiochemical stability.
